# β-secretase inhibitor; a promising novel therapeutic drug in Alzheimer’s disease

**DOI:** 10.3389/fnagi.2014.00165

**Published:** 2014-07-21

**Authors:** Kelly Willemijn Menting, Jurgen A. H. R. Claassen

**Affiliations:** Department of Geriatric Medicine and Radboud Alzheimer Center, Radboud University Medical CenterNijmegen, Gelderland, Netherlands

**Keywords:** Alzheimer’s disease, amyloid cascade hypothesis, APP, amyloid-β, β-secretase, β-secretase inhibitors, BACE1, drug

## Abstract

Alzheimer’s disease (AD) and vascular dementia are responsible for up to 90% of dementia cases. According to the World Health Organization (WHO), a staggering number of 35.6 million people are currently diagnosed with dementia. Blocking disease progression or preventing AD altogether is desirable for both social and economic reasons and recently focus has shifted to a new and promising drug: the β-secretase inhibitor. Much of AD research has investigated the amyloid cascade hypothesis, which postulates that AD is caused by changes in amyloid beta (Aβ) stability and aggregation. Blocking Aβ production by inhibiting the first protease required for its generation, β-secretase/BACE1, may be the next step in blocking AD progression. In April 2012, promising phase I data on inhibitor MK-8931 was presented. This drug reduced Aβ cerebral spinal fluids (CSF) levels up to 92% and was well tolerated by patients. In March 2013 data was added from a one week trial in 32 mild to moderate AD patients, showing CSF Aβ levels decreased up to 84%. However, β-site APP cleaving enzyme 1 (BACE1) inhibitors require further research. First, greatly reducing Aβ levels through BACE1 inhibition may have harmful side effects. Second, BACE1 inhibitors have yet to pass clinical trial phase II/III and no data on possible side effects on AD patients are available. And third, there remains doubt about the clinical efficacy of BACE1 inhibitors. In moderate AD patients, Aβ plaques have already been formed. BACE1 inhibitors prevent production of new Aβ plaques, but hypothetically do not influence already existing Aβ peptides. Therefore, BACE1 inhibitors are potentially better at preventing AD instead of having therapeutic use.

## Introduction

Dementia is a collective name for progressive degenerative brain syndromes which alter memory, behavior, thinking and emotion (WHO, 2012). Several conditions are capable of causing dementia symptoms, as a result of pathological changes in the brain due to the loss of neurons. The most prevalent condition is Alzheimer’s disease (AD); together with vascular dementia, it is responsible for up to 90% of dementia cases (WHO, 2012). According to the WHO, a staggering number of 35.6 million people were diagnosed with dementia worldwide in 2010, and this number is projected to nearly double every 20 years (WHO, 2012). Besides patients, their families and caregivers are also affected. In 2010, the total worldwide costs of dementia were estimated at $604 billion (Wilmo and Martin, 2010). Therefore, blocking disease progression or preventing dementia, and more specifically AD, is desirable for both social and economic reasons. Current therapies, however, are only capable of temporarily slowing down the cognitive decline of AD (Cole and Vassar, 2007). Moreover, successful treatments that address the underlying pathologic mechanisms of AD are lacking (Cole and Vassar, 2007).

This review discusses the development of a high potential therapeutic drug in the battle against AD: the β-secretase inhibitors. The role of β-secretase in the development of AD, the working mechanism of β-secretase inhibitors in general and the potential hurdles in the development of inhibitory drugs will be presented.

### Evidence for the amyloid cascade hypothesis

**Figure 1:**
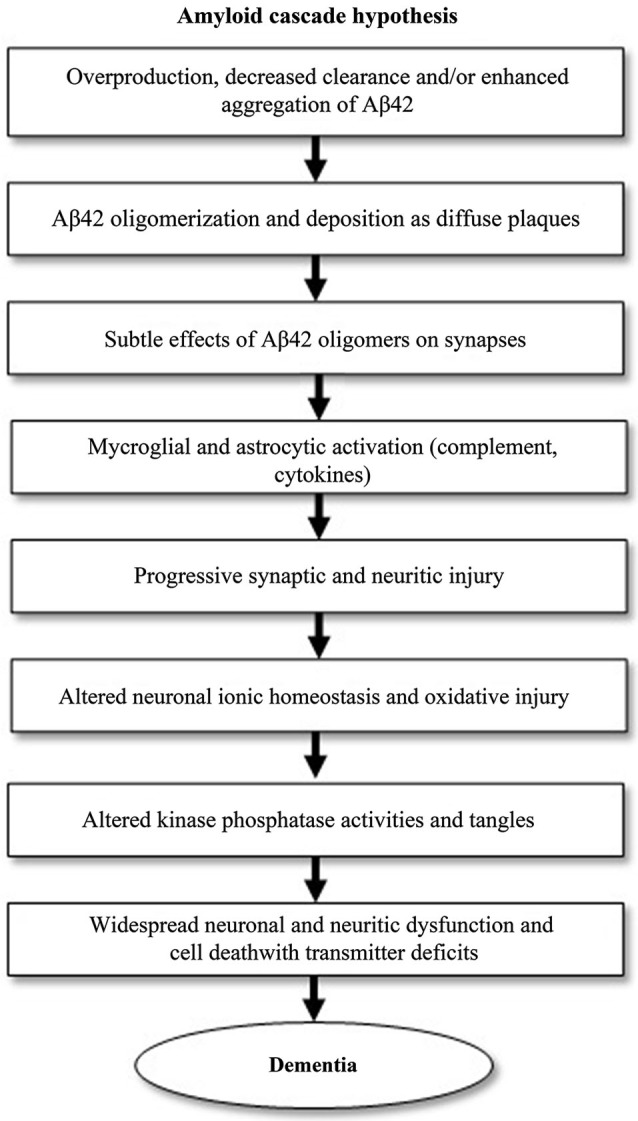
**The amyloid cascade hypothesis, adapted from Citron (2004).** The pathogenic events shown can eventually lead to Alzheimer’s disease (AD). The cascade starts with the generation of amyloid-β42 (Aβ42).

Much of AD research has been focused on the amyloid cascade hypothesis, which was first established by Selkoe and his research group in 1991 (Selkoe, 1991). It postulates that AD is caused by changes in Aβ stability and aggregation or altered amyloid precursor protein (APP) expression, resulting in a chronic imbalance between Aβ production and clearance. Accumulation of aggregated Aβ initiates a cascade that includes inflammatory changes, formation of neurofibrillary tangles and neurotransmitter loss (Citron, 2004; Golde et al., 2006). This cascade is visualized in Figure 1. Blocking the production of Aβ by inhibiting the first proteases required for its generation, β-secretase, may prove to be the next step in blocking AD progression or even preventing the disease.

Several genes, involved in increasing Aβ production or depositioning, have also been implicated in the etiology of AD. These genes include APP, the precursor of Aβ, PSEN1/PSEN2, that code for presenilin proteins which are subcomponents of γ-secretase, and ApoE-ɛ4, an isoform of lipoprotein which is less efficient in catalyzing the breakdown of Aβ compared to other isoforms (Citron et al., 1992, 1997; Schmechel et al., 1993). For example, Down’s syndrome patients who have an extra copy of the APP gene due to a third copy of chromosome 21, have increased Aβ production from birth and develop classical AD pathology by the age of 50. The earliest Aβ depositions are detected at age 12, even before other AD lesions like neurofibrillary tangles are visible (Lemere et al., 1996).

APP transgenic mouse models recapitulate many aspects of the AD pathology, such as Aβ aggregation and cognitive deficits (Ohno et al., 2004). A transgenic mouse model that is unable to produce Aβ (i.e., BACE^−/–^, TG2576^+^ mice), shows improved cognitive function and absence of neuronal loss in comparison to mice that overexpress human APP-695 (i.e., Swedish familial mutation Tg2576^+^) (Ohno et al., 2004; Laird et al., 2005).

In addition, very interesting is the recent finding of Jonsson et al. (2012) of a single nucleotide polymorphism in the APP gene near the β-cleavage site, called A673T, in which alanine 673 is substituted for threonine. This substitution reduces the Aβ levels by approximately 40% in human cell-lines while also protecting carriers of this mutation against AD and cognitive decline (Jonsson et al., 2012). The A673T mutation was found to be significantly more prevalent in the healthy control group than the AD group. Furthermore, mutation carriers performed better on cognitive tests than healthy non-carriers (Jonsson et al., 2012). Another known mutation at exactly the same nucleotide which substitutes alanine for valine (i.e., A673V), achieves the opposite of the A673T mutation: it increases amyloid-β production and is known to cause familial AD (Jonsson et al., 2012).

Data on the A673T mutation, the mouse models and Down syndrome, provide evidence for the amyloid cascade hypothesis *in vivo* and indicate that strategies to lower Aβ levels in the brain are likely to be beneficial in treating AD. Logically, reducing Aβ levels in the brain has been an approach in the development of AD drug treatments for years.

Aβ homeostasis in the brain depends on its production, degradation, efflux out of the brain and the potential formation of insoluble aggregates. These factors can be clinically manipulated to achieve a reduction in Aβ levels in the brain. Current technologies however are incapable in effectively manipulating the degradation and efflux of the peptide. Inhibition of Aβ production is a more promising option for the development of AD drug treatment. Furthermore, by inhibiting the production of Aβ, the Aβ peptide overload can be effectively reduced.

## Amyloid-beta and beta-secretase

### Amyloid-beta biosynthesis

Amyloid-β is produced after the cleavage of APP by two aspartic proteases, β-secretase and γ-secretase (Ghosh et al., 2012). β-secretase cleaves APP at the β-site and is therefore referred to as the “β-site APP cleaving enzyme 1” (BACE1; Vassar et al., 1999). However, β-secretase competes with α-secretase for the APP substrate, and subsequent cleavage by α-secretase does not generate Aβ at all. Accordingly, γ-secretase is a multiprotein complex, containing presenilin, nicastrin, Aph1 and Pen2 and cleaves APP at the γ-site (Wolfe et al., 1999).

**Figure 2:**
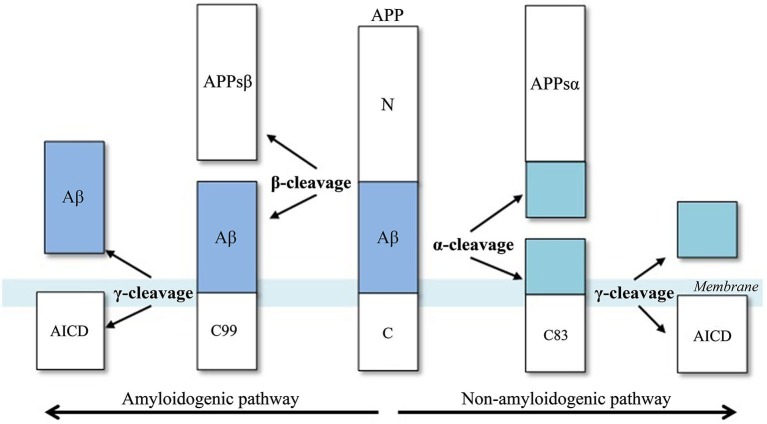
**Aβ-peptide biosynthesis, adapted from Cole and Vassar (2007).** APP is sequentially cleaved by BACE1 (β-secretase) and γ-secretase to generate Aβ. Aβ formation is prevented by the activities of α-secretase, which cleaves APP to generate the secreted ectodomain, APPsα and membrane bound fragment, C83.

Aβ biosynthesis starts when BACE1 cleaves APP at the Asp+1 residue of the Aβ-sequence, which gives rise to the N-terminus of the novel peptide (Figure 2). This cleavage results in two fragments, the secreted ectodomain (APPsβ) and the membrane-bound carboxyl terminal fragment (C99). Next, C99 is cleaved by γ-secretase, forming the C-terminus of Aβ protein and the APP intracellular domain (AICD). This cleavage is not very specific as most Aβ peptides that are generated by γ-secretase activity end at amino acid 40 (Aβ40), while the remainder ends at residue 42 (Aβ42). An excess of the latter peptide is implicated in AD.

Evidently, γ-secretase is a suitable protease for the development of inhibitor drugs for AD and has been actively pursued for years (Ghosh et al., 2012). Several compounds have progressed to human clinical trials. However, γ-secretase has many physiological functions in regulating cell growth and catabolizing membrane proteins fragments. Subsequently, this enzyme is not specific to APP, but also other substrates, including the trans-membrane receptor and signaling protein Notch. Reducing Notch activity interferes with cellular proliferation and differentiation (Walker and Rosen, 2006). Therefore, many proposed γ-secretase inhibitors are toxic for patients, especially if there are no other pathways that can compensate for the loss of γ-secretase. Several γ-secretase inhibitors however have been successful to some extent. The drug R-flurbiprofen (Flurizan^TM^) for example, selectively lowers Aβ42 production through allosteric modulation of γ-secretase activity and does not inhibit the activity of Notch and other substrates (Eriksen et al., 2003). This drug, however, was found to have limited effect in mild AD patients and no effect in moderate AD patients (Green et al., 2009).

This leaves another important protease in the generation of Aβ peptide suitable for developing inhibitor drugs: β-secretase or BACE1. Given that BACE1 is the initiating enzyme in Aβ generation and therefore rate-limiting, it is a prime target for drug development in AD.

### Beta-secretase, the aspartic protease—BACE1 and BACE2

BACE1 is an aspartic protease, which is a family of protease enzymes that use an aspartate residue for the catalysis of their substrates. The 501 amino acid sequence of BACE1 has an N-terminal signal sequence of 21 amino acids (amino acid 1–21), which is the first part of the protein that exits the ribosome during translation (Cole and Vassar, 2007). Together with the pro-peptide domain (amino acids 22–45), the N-terminal signal sequence is removed after translation, forming the mature aspartic protease, which starts at amino acid Glu-46. Near its C-terminus, BACE1 contains a single trans-membrane domain (amino acids 455–480) which is characteristic for mammalian aspartic proteases (Dash et al., 2003).

Furthermore, BACE1 has six luminal cysteine residues that form three intermolecular disulfide bonds and N-linked glycosylation sites (Cole and Vassar, 2007). Glycosylation is a post- and co-translational modification in which glycans are attached to the protease. BACE1 also contains two active site aspartate residues in its extracellular protein domain (amino acids 93–96 and 289–292), which are necessary for the protease activity of BACE1 (Hussain et al., 1999). This active site is perfectly positioned for cleaving APP at the β-site.

BACE1 is not only regulated at the translational level, but also at transcriptional level (Roßner et al., 2006). The upstream promoter, a region of DNA that initiates transcription and is located upstream of the transcribed gene, lacks the typical TATA box, a general transcription factor binding site. However, there are other transcription factor binding sites (such as for CREB and KF-κB) that are known to influence the transcriptional activity of the BACE1 promoter.

**Figure 3:**
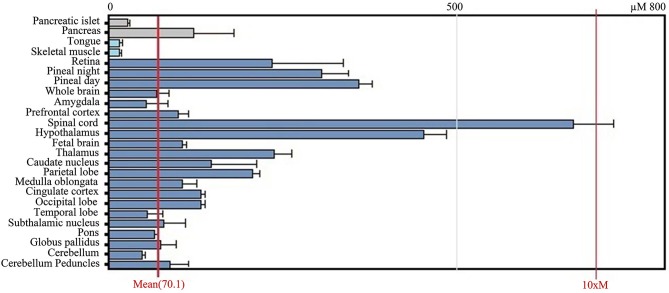
**GeneAtlas BACE1 mRNA expression pattern in human tissues, adapted from BioGPS.org [accessed on 2014-04-07]**. The BACE1 mRNA is predominantly expressed in brain tissues such as the spinal cord and hypothalamus, but also in the pancreas.

After transcription, the BACE1 mRNA is transported out of the nucleus and enters the cytoplasm where it is translated. Tissue culture and animal studies indicate that BACE1 is expressed in all tissues, but mRNA levels are highest in the pancreas and in the brain (both in brains affected and unaffected by AD; Figure 3). The relatively high levels of BACE1 found in the pancreas indicate a (β-secretase) function of BACE1 in the pancreas. However, research shows that BACE1 mRNA in the pancreas encodes a shortened splice variant that is deficient in β-secretase activity (Bodendorf et al., 2001). This is consistent with high BACE1 mRNA levels in the pancreas, but low β-secretase activity in the organ. However, the precise function of this pancreas specific BACE1 variant remains unknown.

When BACE1 is transfected in APP-overexpressing cell-lines, the protease induces an immense increase in β-secretase activity. In comparison to control cells, the products of β-secretase cleavage (APPsβ and C99) are dramatically increased (Vassar et al., 1999). Indicative of the competition between β- and α-secretase, APPsα levels are reduced. In accordance with these findings, Aβ peptide levels are elevated (Vassar et al., 1999). Furthermore, when APP-overexpressing cells are exposed to BACE1 antisense oligonucleotides, BACE1 mRNA levels are decreased and β-secretase activity is inhibited (Vassar et al., 1999). Several other studies indicate that BACE1 expression is increased in the brains of AD patients when compared to healthy subjects (Fukumoto et al., 2002; Holsinger et al., 2002).

Immediately after the discovery of BACE1, a second aspartic protease was identified that has 64% amino acid sequence similarity to BACE1 and also contains a C-terminal trans-membrane domain. This protease has received much interest, as it is located in the Down syndrome region of chromosome 21. Like BACE1, it could have β-secretase activity (Saunders et al., 1999). However, studies indicate that BACE2 is primarily expressed peripherally and brain expression is extremely low (Bennett et al., 2000). Also, antisense inhibition of BACE2 does not reduce Aβ generation while overexpression of BACE2 does not result in an increased Aβ production (Yan et al., 1999; Bennett et al., 2000). Recent studies show that overexpression of BACE2 actually reduces Aβ production in neurons of the mutant Tg2576 mice (i.e., mice that overexpress APP) (Hong et al., 2002; Sun et al., 2006).

Data clearly demonstrates that BACE2 has the potential to cleave APP within the Aβ domain, but is not involved in AD pathogenesis. Instead, the decrease of Aβ production in APP mutant mice under the influence of BACE2 overexpression may indicate therapeutic interventions of BACE2 in preventing AD pathogenesis (Sun et al., 2006).

### BACE1 knockout studies

Evidently, the discussed data suggests a critical role for BACE1 in Aβ peptide generation and indicates its suitability as target for therapeutic drug inhibition. Further research involves knock-out (KO) models, which are important in the development of inhibitory drugs. A natural occurring, partial BACE1 KO in humans is the previously discussed A673T mutation in the Icelandic population (Jonsson et al., 2012).

The first findings by several independent groups on BACE1 KO mice models show that these animals do not produce cerebral Aβ peptides and amyloid deposits, and remain healthy and fertile (Ohno et al., 2004). Swedish APP-overexpressing mice (i.e., Tg2576^+^) have highly elevated Aβ peptide levels in the brain and develop memory deficits and plaques in later life. Crossing Tg2576 mice with BACE1 KO mice creates a new mouse model (i.e., BACE1^−/–^, Tg2576^+^) that do not generate Aβ, APPsβ and C99 (Luo et al., 2001). Moreover, all products that are generated through β-secretase activity are non-existent in these BACE1 KO mice, even when APP is over expressed. Subsequently, several studies were conducted to determine whether the lack of Aβ due to BACE1 deficiency also prevents memory deficits in Tg2576 mice. BACE1^−/–^, Tg2576^+^ mice indeed do not develop memory deficits in comparison to Aβ-overproducing mice (Kuo et al., 1996; Ohno et al., 2004). This data unanimously demonstrates that BACE1 is responsible for the β-secretase cleavage and no compensatory mechanisms are present in these mice models (Cai et al., 2001; Luo et al., 2001; Roberds et al., 2001).

However, complete elimination of BACE1 may have unintended side effects similar to what was demonstrated in studies on γ-secretase inhibition (Walker and Rosen, 2006). Initial research indicates that the absence of BACE1 does not affect the development of embryo’s and the physiology, biochemistry, morphology and behavior of adult KO mice. Further and more detailed analyses however show that complete deletion of BACE1 activity does have effects (Cole and Vassar, 2007): BACE1 KO mice have abnormalities in both spatial and reference memories and impairments in temporal associative memory. They do behave normal in social recognition. These findings suggest that BACE1 not only functions in Aβ production, but also in normal memory processing within the hippocampus (Cole and Vassar, 2007).

In addition to the memory defects found in BACE1 KO mice, BACE1 inhibition might also cause demyelinating side effects. In 2006 it was discovered that neuregulin-1 is a BACE1 substrate and is important for myelination early in life (Hu et al., 2006; Willem et al., 2006). However, treatment with BACE1 inhibitors will only take place in later life and will therefore probably not cause demyelination. Furthermore, biochemical analysis indicate that BACE1 processes the neuregulin-1 isoform Ig-containing β1 (IgNrg1β1; Cheret et al., 2013). Graded reduction in the IgNrg1 isoform signal strength *in vivo*, correlates with increasingly severe deficits in the formation and maturation of muscle spindles, which are critical for muscle coordination (Cheret et al., 2013). Also, through pharmacological inhibition and mutagenesis in animals it was found that BACE1 and Nrg1 are essential to maintain motor coordination. Therefore, a partial inhibition of β-secretase activity seems most beneficial. Furthermore, to evaluate potential toxicity arising from the inhibition of BACE1, studies need to be conducted to determine (additional) BACE1 substrates *in vivo* (Vassar and Kandalepas, 2011).

## Beta-secretase inhibitors

### Hurdles in the development of the first beta-secretase inhibitors

The previous data concerning BACE1 knockout studies, overexpression and antisense inhibition all support the hypothesis that BACE1 is a prime drug target for inhibiting the production of Aβ. The protease is critical in the biosynthesis of Aβ and found to be elevated in the brains of AD patients (Fukumoto et al., 2002; Holsinger et al., 2002). However, complete abolishment of BACE1 activity may result in harmful phenotypical defects in memory processing, myelination and motor coordination.

Another hurdle that has to be overcome in the development of a potent β-secretase inhibitor, is the blood-brain barrier (BBB). This is the separation of circulating blood from extracellular fluid in the central nervous system. The endothelial cells responsible for this barrier restrict diffusion of large or hydrophilic molecules and other objects into the brain. Passing the BBB is essential, as BACE1 cleaves APP in the endosomes of neurons within the brain. Therefore, a β-secretase inhibitor needs to cross the BBB and neuronal membranes. Endothelial cells do allow the diffusion of hydrophobic and small molecules like hormones and oxygen. The largest molecular size that can cross the BBB is approximately 550 DA (Ghosh et al., 2012).

### The development of beta-secretase inhibitors

An enzyme inhibitor is a molecule that binds to the enzyme and thereby decreases its activity. The binding of the inhibitor hinders the enzyme from catalyzing a reaction. The binding of an inhibitory drug can either be irreversible or reversible. Irreversible inhibitors covalently bond with amino acid residues that are needed for the enzymatic activity, while reversible inhibitors bind non-covalently to either the enzyme itself or the enzyme/substrate complex, through hydrogen bonds, ionic bonds or hydrophobic interactions. There are four different kinds of reversible enzyme inhibitors (Berg et al., 2002):

- Competitive inhibition: the inhibitor has affinity for the active site of an enzyme where the substrate also binds. This leads the substrate and the inhibitor to compete for access to the enzyme’s active site. Competitive inhibitors often mimic the structure of the natural substrates. Conversely, sufficiently high concentrations of the natural substrate, can out-compete the inhibitor and reduce its effects.
- Uncompetitive inhibition: the inhibitor binds to the enzyme/substrate complex, hindering the catalysis of the natural substrate.
- Mixed inhibition: when the inhibitor binds to the enzyme, it affects the enzyme’s binding to the substrate and vice versa. It is possible for these inhibitors to bind at the active site, but inhibition generally occurs from an allosteric effect where the inhibitor binds adjacent to the active site, changing the conformation of the enzyme. This results in reduced affinity of the substrate for the active site.
- Non-competitive inhibition: binding of the inhibitor to the enzyme reduces enzyme activity, but does not affect the binding of a substrate to the active site. The concentration of the inhibitor determines the extent of inhibition.

As previously discussed, BACE1 is a membrane bound aspartic protease. Traditionally, protease inhibitors are developed by natural product screening for lead substrates by substrate-based methods and subsequent optimization (West and Fairlie, 1995). Such optimization includes the replacement of hydrolyzable peptide bonds by non-hydrolyzable isosteres. A peptide bond is a chemical bond formed between two amino acids when the carboxyl group of one reacts with the amino group of the second amino acid. With the addition of water, this chemical peptide bond can be cleaved in a reaction that is referred to as hydrolysis. Isosteres are defined as functional groups that mimic peptide bond hydrolysis, but cannot be cleaved by the aspartic proteases (Dash et al., 2003). Furthermore, these stable structures are thought to bind more tightly to the aspartic protease than natural substrates. Studies on the aspartic protease inhibitor pepstatin indicates that this increased affinity is not only due to imitation of the transition state of peptide bond hydrolysis, but also due to the deletion of the water molecule (hydrogen) that is bonded to the aspartate residues (Dash et al., 2003).

Inhibitors bind to BACE1 through non-covalent interactions and are therefore reversible inhibitors (Dash et al., 2003). Inhibition depends on BACE1 having a greater affinity for the inhibitor than for APP, so this is a form of competitive inhibition. Maximal inhibitor affinity is created by increasing the number of non-covalent interactions between BACE1 and the inhibitor. Aspartic proteases like BACE1 have a catalytic domain containing a pair of active-site aspartic acid residues. Furthermore, BACE1 has an elongated substrate-binding site, the subsites, that can bind up to 11 amino acids of substrates. These substrates are processed with the aid of the two aspartic acid residues in the active-site (Dash et al., 2003; Ghosh et al., 2012). Inhibitor specificity for BACE1 can be built by taking advantage of the collective interactions between a putative inhibitor on both sides of its peptide bond and a part of the substrate-binding groove of BACE1 (Dash et al., 2003). The 11 subsites have a broad amino acid preference, but many central ones (such as P1 and P1′) prefer hydrophobic side chains. This characteristic can be utilized in the development of inhibitors, as good lipophilicity is important for membrane penetration and passing the BBB (Ghosh et al., 2012).

### The evolution of beta-secretase inhibitors

The first reported BACE1 inhibitor is the P10-P4′ StatVal, developed by Elan Pharmaceuticals and it is a substrate analog that substitutes P1-(S)-statine (Sinha et al., 1999). Shortly thereafter, a new BACE1 inhibitor was developed and named OM99-2, an 8-residue transition analog that was designed based on substrate specificity (Ghosh et al., 2000; Lin et al., 2000). The scissile peptide bond of the substrate was replaced by a Leu-Alahydroxyethylene transition-state isostere. The crystal structure of BACE1/OM99-2 complex, however, indicated that the S_3′_ and S_4′_ subsites were not correctly formed. Despite its excellent inhibitory potency *in vitro*, the peptidic structure inhibited application *in vivo* (Luo and Yan, 2010).

Further research into the subsite preference of BACE1 and binding residues led to the design of a second generation of β-secretase inhibitors: OM00-3 (Hong et al., 2002). The interactions between the P1/P1′ subsite region of OM00-3 and the substrate binding cleft of BACE1 are the same as for OM99-2 (Hong et al., 2002). The multiple interactions between Arg-307 amino acid of BACE1 and carboxylate oxygen atoms of P4 Glu amino acid of the inhibitor, contributes to the inhibitor binding. Additionally, leucines at P3 and P1 of OM00-3 interact with the S_3_ site of BACE1 and with each other. This stabilizes the conformation of the inhibitor.

Many substrate-based peptidomimetic inhibitors are developed by academic research groups and pharmaceutical companies. The problem with the BACE1 inhibitors P10-P4′, OM99-2 and OM00-3, is that they do not have the qualified drug properties: they are either too large in size, have a too short half-life *in vivo*, are not capable of passing the BBB or have low oral availability (Luo and Yan, 2010). Therefore, later generations of BACE1 inhibitors are smaller and often non-peptidic.

In 2008, the pharmaceutical company CoMentis revealed that it developed a new potent BACE1 inhibitor that passed the phase 1 clinical trial (Hey et al., 2008; Koelsch, 2008; Ghosh et al., 2012). This small compound is named CTS-21166, can pass the BBB, has high oral availability and is both selective and stable. CoMentis left its structure undisclosed, but revealed that by injecting CTS-21166 into an APP transgenic mouse (expressing both Swedish and missense London mutations) the Aβ peptide and plaques levels are reduced by 35% and 40% respectively (Luo and Yan, 2010). Also, the drug did not damage the myelin sheath of the mouse model, indicating that BACE1 indeed only aids myelination during early development, as suggested previously (Willem et al., 2006). Data from the human phase I study indicates that CTS-21166 is safe when injected intravenously into AD patients with doses as high as 225 mg. Results show a dose-dependent reduction of plasma Aβ levels for an extended period of time (Strobel, 2008). Significant inhibition of plasma Aβ persisted beyond 72 h (Ghosh et al., 2012). Similar results were obtained from a second phase I trial on subjects receiving an oral liquid solution of 200 mg CTS-21166 (Ghosh et al., 2012).

The news of CTS-21166 led another pharmaceutical company, Merck, to consider phase I testing with their BACE1 inhibitor (Luo and Yan, 2010). In April 2012, Merck presented their phase I data on the 64th American Academy of Neurology (ANN) annual meeting (Forman et al., 2012). In 40 healthy adults single doses of 100 or 500 mg of their inhibitor MK-8931 reduced Aβ peptide concentration levels in cerebral spinal fluids (CSF) up to 92%. In the multiple dose study, CSF Aβ peptide levels dropped 50% and 80% for 10 and 40 mg doses. The drug was well tolerated, as no serious adverse events or study discontinuations occurred. Furthermore, due to the 20 h half-life of MK-8931 it is ideal for once-a-day dosing (Forman et al., 2012; Landhuis, 2012).

At the end of 2012 it was announced that Merck initiated the Phase II/III studies of MK-8931 for the treatment of AD. In these tests the safety and efficacy of MK-8931 versus placebo are determined in patients with mild and moderate AD. The global study is referred to as EPOCH and is a 78-week phase II/III clinical trial in which doses of 12, 40 or 60 mg are tested. The phase III trial will assess change from baseline in cognition and function using several tests, but also AD biomarkers, such as CSF total tau, hippocampal volume and brain amyloid. MK-8931 is the first BACE1 inhibitor to advance to this stage of clinical research, with 200 participants in phase II and 1800 participants in phase III. At the 11th international conference on AD and Parkinson’s Disease that was held in the beginning in March 2013 in Florence, Merck added the first data from a trial that lasted a week in 32 mild to moderate AD patients of the mean age of 73 years (Bowman Rogers and Strobel, 2013; Forman et al., 2013; Dobrowolska et al., 2014). Results indicate a decrease in CSF Aβ40 and Aβ42 of up to 84% for the 12, 40 or 60 mg doses. This indicates that that the presence of high cerebral amyloid concentrations does not change the pharmocodynamic and pharmacokinetic properties of this BACE1 inhibitor. The exact working mechanisms and molecule structures of BACE1 inhibitors MK-8931 and CTS-21166, however, remain intellectual property and are unfortunately inaccessible.

Another BACE1 inhibitor that has reached phase II trials is the Eli Lilly’s inhibitor LY2886721. The data on phase I trial were first presented at the Alzheimer’s Association International conference in 2012. Daily dosing during 2 weeks, reduced BACE1 activity by 50–75% and CSF Aβ42 by 72% (Willis et al., 2012; Bowman Rogers and Strobel, 2013). Recently, Lilly reported that the phase II trial of LY2886721 was terminated due to liver abnormalities that were found in 4 out of 45 patients (Rogers, 2013). This toxicity, however, does not have to be related to the working mechanism of the inhibitor, but can represent off-target effects as the livers of BACE1 knockout mice are normal.

During the Alzheimer’s Association International conference in 2012 another BACE1 inhibitor was presented: E2609. In a phase I study, healthy young subjects aged 30–55 received single doses of 5–800 mg, while healthy elderly subjects aged 65–85 received a single dose of 50 mg. Results show that E2609 was well-tolerated across all doses, and causes prolonged reduction of plasma Aβ of up to 45% (Lai et al., 2012, 2013). Up until now no updates are announced on this inhibitor, but more thorough clinical evaluation of E2609 is underway.

## Discussion

Research on KO, overexpression and antisense inhibition of BACE1 show that BACE1 is the primary protease responsible for the production of Aβ within the brains of healthy patients and diseased patients. Especially the recent findings that the A673T mutation in the APP gene results in a BACE1 activity reduction and protects subjects against AD, seems very promising. Previous efforts concerning the development of drugs based on the amyloid cascade hypothesis, were mainly focused on another aspartic protease, γ-secretase. BACE1, however, seems more potent as a drug target as it is the first protease in the Aβ biosynthesis and therefore is possibly rate limiting.

Over recent years, research groups and companies have tried to develop the first potent BACE1 inhibitor. Due to difficulties in developing a potent protease inhibitor, many have not reached the clinical trials stage. Recently however, pharmaceutical company Merck and co. announced that it developed an inhibitory drug for the treatment of AD, named MK-8931. At the end of 2012 this drug became the first BACE1 inhibitor to reach phase II/III of clinical human trials. The latest results from pharmaceutical companies indicate that BACE1 inhibitors are capable of reducing Aβ peptide levels in the brain, suggesting that they may be effective in treating AD.

BACE1 inhibitors however still have a long way to go before they are applicable in the battle against AD. First, extreme reductions in Aβ peptide levels through the inhibition of BACE1 may have harmful side effects. Furthermore, it was discovered that neuregulin-1, a peptide that is essential for the normal development of the nervous system and the heart, is also a substrate of BACE (Britsch, 2007) and a complete abolishment of BACE1 could affect the myelination of neurons, due to the functioning of neuregulin-1. However, BACE1 inhibitor CTS-21166, produced by pharmaceutical company CoMentis, did not influence the myeline sheath in their mouse model. Apparently BACE1 substrate neuregulin-1 only influences the myeline sheath during early development, although it should still be taken into account that complete inhibition of BACE1 may influence memory capacity or have other side effects. Recent research suggests that BACE1 has more additional substrates and that it is involved in forming connections in the brain (Kuhn et al., 2012; Zhou et al., 2012). The study conducted by Vassar indeed indicates that BACE1 KO mice have axon guidance defects (Rajapaksha et al., 2011). These data however should be translated to human data, and it is unknown how serious these defects will be. Future studies on BACE1 substrates should evaluate the potential toxicity of BACE1 inhibitors *in vivo* (Vassar and Kandalepas, 2011).

Furthermore, no single BACE1 inhibitor has currently passed phase II/III of clinical trials and possible side effects in Alzheimer patients and the exact clinical efficacy of the inhibitors remain unknown. However, with the announcement that the inhibitory drug presented by Merck and co. has reached phase II/III, new data on possible side effects and the exact clinical efficacy are therefore within reach. In the meantime, phase I data indicate that there are very few and mild side effects.

In addition to the concern about possible side effects, there remains doubt about the clinical efficacy of BACE1 inhibitors. Results on the γ-secretase inhibitor R-flurbiprofen show that it has limited effect in mild AD patients and no effect in moderate AD patients (Green et al., 2009). This could also apply to BACE1 inhibitors, as both are aspartic proteases and components of a cascade responsible for the production of the Aβ peptide. It must be taken into account that BACE1 inhibitors prevent the production of new Aβ peptides and plaques, but do not influence already existing Aβ peptides. In moderate AD patients, Aβ peptides have already substantially accumulated and many plaques are formed. Therefore, BACE1 inhibitors are potentially better at preventing AD instead of having a therapeutic use.

## Conflict of interest statement

The authors declare that the research was conducted in the absence of any commercial or financial relationships that could be construed as a potential conflict of interest.
